# Multivariate analysis of seed chemical diversity among wild fenugreek (*Trigonella monantha* C. A. Mey.) ecotypes

**DOI:** 10.1186/s12870-023-04327-3

**Published:** 2023-06-16

**Authors:** Neda Esamaeilinejad Hasaroeih, Farangis Ghanavati, Foad Moradi, Jahangir Abbasi Kohpalkani, Majid Rahimizadeh

**Affiliations:** 1 Department of Agronomy, Faculty of Agriculture, Bojnourd Branch, Islamic Azad University, Bojnourd, Iran; 2 Seed and Plant Improvement Institute, Agricultural Research, Education and Extension Organization, Karaj, Iran; 3 Agricultural Biotechnology Research Institute of Iran (ABRII), Agricultural Research, Education and Extension Organization, Karaj, Iran; 4 Department of Crop Sciences, Faculty of Agriculture, Bojnourd Branch, Islamic Azad University, Bojnourd, Iran

**Keywords:** Chemical diversity, Trigonelline, Medicinal plant, Wild fenugreek germplasm

## Abstract

**Background:**

Wild fenugreek (*Trigonella monantha*), a multi-purpose annual plant, has traditionally been used as a food, forage, and medicinal plant. However, the knowledge of the diversity of its chemical characteristics is limited. In this study, 40 wild fenugreek ecotypes collected from their natural habitats in Iran and grown together in field conditions, were analyzed for their seed chemical properties.

**Results:**

The ecotypes were cultivated in a randomized complete block design (RCBD) with three replications. The results of ANOVA revealed a significant difference among the ecotypes for all measured characters (P < 0.01). The results showed a high level of diversity among the ecotypes based on the measured characters, including antioxidant activity (48.19 to 86.85%), phenol (0.82 to 1.51 mg gallic acid per g dry weight), flavonoid (1.07 to 3.11 mg quercetin per g dry weight), trigonelline (0.02 to 0.08 mmol/l), 4-hydroxyisoleucine (0.197 to 0.906 mg/g), sucrose (0.13 to 3.77 mM), glucose (1.07 to 12.1 mM), and fructose (13.3 to 45.5 mM). The cluster analysis divided the ecotypes into four groups and the PCA analysis showed that the three first components explained 73% of the total variance among the ecotypes. Also, heat map correlation revealed that many positive and negative correlations were observed among the measured characters. The results did not show a relationship between the amounts of compounds and the place of sample collection.

**Conclusions:**

The present study suggests considerable diversity in the seed chemical compositions of the wild fenugreek ecotypes. Therefore, many ecotypes could be useful for medicinal purposes, as well as for human nutrition.

## Background

Medicinal plants have the potential to be effective in preventing and treating various diseases by synthesizing secondary and active ingredients such as polyphenols [1].

The genus Trigonella belongs to the Fabaceae family and comprises over 135 species that are widely distributed worldwide [2]. In Iran alone, more than 32 species can be found [3]. Trigonella species have a long history of use in traditional medicine for the treatment of diabetes, hypercholesterolemia, and inflammation [4]. Fenugreek, a Trigonella species, contains compounds such as diosgenin, 4-hyroxyisoleucine, fenugreekine, and fenugreekoside, which have been shown to have therapeutic effects [5]. Recent clinical studies have demonstrated that fenugreek can improve glycemic control and cholesterol levels in individuals with diabetes [6].

*Trigonella monantha*, commonly known as wild fenugreek, is an annual plant that grows on or slightly above the soil surface and has a somewhat hairy texture. The plant possesses a variety of therapeutic actions, including hypoglycemic [7], antibacterial [8], anti-inflammatory [9], and antioxidant activities [8]. Fenugreek seeds are known for their protective effects on the heart and liver, as well as their ability to treat hyperglycemia (hypoglycemia below 60 mg/dl), hypocholesterolemia, and neurological and hormonal disorders [10]. Recent studies have suggested that fenugreek seeds may also possess anticancer properties, which could be beneficial in preventing and treating various types of cancer [11]. Trigonelline, a physiologically active compound found in these plants, induces leaf movements and accumulates in plants during stress [12]. Studies have shown that trigonelline can reduce blood glucose levels by increasing insulin secretion and improving insulin sensitivity [13]. Additionally, 4-Hydroxy isoleucine, another component found in fenugreek, is involved in reducing blood sugar [14, 15]. The concentration of 4-Hydroxy isoleucine in 100 g of dried fenugreek seeds ranges from 90 to 300 mg [16].

Fenugreek seeds are also known for their high total antioxidant activity due to the presence of various bioactive compounds such as flavonoids, alkaloids, and phenolic acids [11]. Furthermore, fenugreek seeds are also rich in flavonoids, which are known to have potent antioxidant properties. A study conducted on fenugreek leaves found that they contain high levels of flavonoids, such as rutin, quercetin, and kaempferol [17]. Additionally, fenugreek seeds contain high levels of phenolic compounds, including gallic acid, caffeic acid, and chlorogenic acid [18]. These phenolic compounds have been found to possess various health benefits, including antioxidant and anti-inflammatory properties. Fenugreek seeds are also a good source of soluble sugars, such as sucrose, glucose, and fructose. The plant seeds contain high levels of total soluble sugars, which may have potential health benefits, such as improving energy metabolism [19].

Medicinal plants have been a rich source of new leads and inspiration for novel phytochemical components at the forefront of drug discovery [20]. Discovering and identifying new phytochemical and antioxidant compounds and therapeutic properties of plants in inhibiting free radicals is critical in preventing and treating many common inflammatory diseases [21–23]. For centuries, these plants were the primary healthcare for people in developing nations, and 20 to 80% of developing countries’ populations still rely on medicinal plant products [24]. Although thousands of medicinal plant species are estimated to be used traditionally in Iran, only a small percentage of them have been screened for phytochemical components and antioxidant activity [25, 26]. Therefore, the present study aims to assess the phytochemical properties of 40 *T. monantha* ecotypes collected from different regions of Iran.

## Materials and methods

### Plant materials and study location

Forty ecotypes of *T. monantha* were collected from various regions of Iran, and permission was obtained from the Agricultural Research, Education and Extension Organization of Iran to collect plant samples. Voucher specimens were stored at the National Plant Gene-Bank in Karaj, Iran (Table 1). The plants were identified by the corresponding author, Dr. Farangis Ghanavati.

**Table 1:** The locations of 40 *T. monantha* ecotypes collected from different regions of Iran

Code	Origin	Voucher no.	Latitude	Longitude	Altitude
Tm1	Azna, Lorestan	27,893	33 45 N	49 45 E	1861
Tm2	Qom, Qom	17,594	34 64 N	50 87 E	936
Tm3	Bisheh, Lorestan	98	33 33 N	48 87 E	1225
Tm4	Bajestan, Khorasanjonobi	25	34 51 N	58 19 E	1272
Tm5	Kashmar, Khorasanjonobi	26	35 24 N	58 45 E	1051
Tm6	KalateAbdollah, Khorasajonobi	27	35 90 N	60 46 E	910
Tm7	Jajrood, Tehran	32	35 74 N	51 70 E	1484
Tm8	Chitgar, Tehran	33	35 71 N	51 16 E	1206
Tm9	Peykan Shahr, Tehran	35	35 73 N	51 18 E	1264
Tm10	Karaj, Alborz	38	35 82 N	50 96 E	1312
Tm11	Taleghan, Alborz	40	36 17 N	50 76 E	1809
Tm12	Buin Zahra, Ghazvin	43	35 77 N	50 06 E	1213
Tm13	Abyek, Alborz	47	36 04 N	50 53 E	1261
Tm14	Salafchegan, Qom	49	34 47 N	50 45 E	1385
Tm15	Salafchegan, Qom	50	34 56 N	50 43 E	1415
Tm16	Shiraz, Fars	53	29 59 N	52 53 E	1519
Tm17	Maharlu Lake, Fars	55	29 44 N	52 80 E	1461
Tm18	Maharlu, Fars	58	29 35 N	52 81 E	1481
Tm19	Fasa, Fars	59	28 94 N	53 64 E	1380
Tm20	Bamou Park, Fars	69	29 67 N	52 67 E	2008
Tm21	Jaroo, Alborz	124	35 69 N	50 53 E	1415
Tm22	Zarrin Chegha, Lorestan	75	33 64 N	48 25 E	1425
Tm23	Kaka Reza, Lorestan	78	33 72 N	48 25 E	1548
Tm24	Aleshtar, Lorestan	83	33 86 N	48 25 E	1605
Tm25	Zaghe, Lorestan	94	33 49 N	48 70 E	1812
Tm26	Haroo Bridge, Lorestan	95	33 52 N	48 77 E	1754
Tm27	Azna, Lorestan (2)	97	33 45 N	49 45 E	1861
Tm28	Bisheh, Lorestan	99	33 32 N	48 87 E	1228
Tm29	KhoramAbad, Lorestan	102	33 48 N	48 35 E	1188
Tm30	Choqa, Lorestan	107	33 37 N	48 04 E	1164
Tm31	KharmanKuh, Fars (1)	61	29 21 N	53 57 E	3220
Tm32	KharmanKuh, Fars (2)	61	29 21 N	53 57 E	3220
Tm33	Salafchegan, Qom	48	34 64 N	50 87 E	936
Tm34	Bamou Park, Fars (2)	67	29 67 N	52 67 E	2008
Tm35	Sabzpooshan Kuh, Fars	63	29 42 N	52 56 E	1488
Tm36	Rudmajan, Khorasanjonobi (1)	29(1)	35 44 N	58 83 E	1725
Tm37	Rudmajan, Khorasanjonobi (2)	29(2)	35 44 N	58 83 E	1725
Tm38	Kashmar, Khorasanjonobi	30	35 24 N	58 45 E	1051
Tm39	Rudmajan, Khorasanjonobi (3)	29(3)	35 44 N	58 83 E	1725
Tm40	Kaka Reza, Lorestan	79	33 72 N	48 25 E	1548

The ecotypes were grown in a randomized complete block design (RCBD) with three replications in a field at the Seed and Plant Improvement Institute in Karaj, Iran (35°50’ N, 50°56’ E, 1380 m above sea level). The results of soil analysis are presented in Table 2. The mean annual rainfall and temperature of Karaj county are 244 mm and 15 °C, respectively.

**Table 2:** The results of soil analysis

Soil properties	EC (ds.m^− 1^)	pH	Organic matter (%)	Potassium (mg kg^− 1^)	Phosphorus (mg kg^− 1^)	Total nitrogen (%)
	0.88	8.2	1.52	251	14.2	0.128

On April 5th, the seeds of the ecotypes were scarified and sowed. The plants were harvested on September 16th without any application of fertilizers or supplementary feeding. Each ecotype for each replication was cultivated in a plot of 200 by 60 centimeters, with four rows spaced at 50 cm intervals between the rows and 15 cm intervals between the plants. The plants were irrigated once a week using surface and furrow irrigation until one week before harvest. For metabolite analysis, ten plants were randomly sampled from each plot.

### Seed extract

The extraction process was carried out using methanol as the solvent. Fenugreek seed powder (100 g) was mixed with 80% methanol and left to stand at room temperature for 24 h. The resulting extract was filtered through paper and transferred to a flask, then stored at 4 °C to prevent degradation of the compounds prior to chemical analysis.

### Antioxidants activity

The antioxidant content of wild fenugreek seeds was determined by the 1,1-diphenyl-2-picrylhydrazyl (DPPH) free radical method. The experiment was based on Brand-Williams et al.’s [27] method with minor modifications. The percentage of Radical scavenging activity of the wild fenugreek seed extracts was calculated based on the following formula.

1 $$DPPH{\text{ }} = {\text{ }}100{\text{ }} \times {\text{ }}\left( {AC - AS} \right){\text{ }}/{\text{ }}AC$$

Whereas, AC and AS are the absorbance of the control and the test solution, respectively. The numbers obtained are equal to the percentage of free radical scavenging in the methanolic extract of the ppm of the samples [28].

### Flavonoids content

To determine the flavonoid content, the methanolic extract (0.5 ml) was mixed with 1.5 ml of methanol and 0.1 ml of 10% aluminum chloride (w/v) in ethanol. This was followed by the addition of 0.1 ml of 1 M potassium acetate and 2.8 ml of distilled water [29]. The mixture was incubated at room temperature for 30 min and the absorbance was measured at 415 nm. The standard curve was prepared using different concentrations of quercetin, and the amount of flavonoids equivalent to quercetin per gram of seed dry weight was determined.

### Total phenol content

The distilled water (1.16 ml) along with 100 µl of Folin-Ciocalteu were added to 20 µl of methanolic extract and then 300 µl of 1 M sodium carbonate (10.6 g per 100 ml of distilled water) [30]. The mixture kept in the water bath for 30 min at 40 ºC. Finally, the absorbance was measured at 760 nm. The standard curve was calculated based on gallic acid with different concentrations. The amount of phenolic compounds equivalent to gallic acid per gram of dry powder was measured.

### Trigonelline

The concentration of trigonelline in fenugreek seeds was determined using a modified version of the method described by Rajabi Hashjin et al. [31]. First, the seeds were pulverized and extracted with methanol, and the upper phase was collected, evaporated, and stored until HPLC analysis was performed. The HPLC analysis was conducted using an Agilent 1260 Infinity device and an aminex column, with a mobile phase consisting of methanol and water. Trigonelline content was determined by comparing the peak area with that of a Merck trigonelline standard.

### 4-hydroxy isoleucine

In this study, we used the ortho-phthalaldehyde (OPA) derivatization and HPLC method to quantify 4-hydroxyisoleucine, following the procedure described by Hajimehdipoor et al. [32]. The method involved preparing various concentrations of 4-hydroxyisoleucine in distilled water, derivatizing the amino acids with OPA, and separating and quantifying the amino acids using HPLC with a linear gradient mobile phase.

### Soluble sugars (sucrose, glucose, and fructose)

The method used in this study involved extracting soluble sugars from the sample using ethanol. Barium hydroxide and zinc sulfate were added to the extract to separate the sugars from other compounds [33]. The separated sugars were then subjected to HPLC analysis, which was used to quantify individual sugars. The RI detector was employed to detect the sugars.

### Statistical analysis

Statistical analyses, including analysis of variance and LSD mean comparison, as well as PCA, were conducted using SAS software version 9.4 (SAS Institute Cary, NC, USA, 1988). Cluster analysis was performed using the UPGMA method. The heat map clustering, correlation analysis, and PCA biplot were visualized using MetaboAnalyst [34].

## Results and discussion

After conducting ANOVA, significant differences were observed among the ecotypes for all measured parameters (P < 0.01; Table 3). These findings suggest a high level of variation among the ecotypes, which could be attributed to both genetic and environmental factors [21, 22]. Previous research by Riasat et al. [35] also reported considerable variations in the morphology and polyphenolic composition of fenugreek species, including *T. uncata*, *T. persica*, *T. elliptica*, *T. monantha*, *T. foenum graecum*, and *T. coerulescens*. Moreover, Tunisian fenugreek cultivars were found to exhibit significant morphological and chemical variations [36] which is consistent with our current results. In our previous study, significant differences in morphological characteristics and seed yield were also observed among genotypes [37].

**Table 3:** The results of ANOVA for the measured characters

S.O.V	df	Antioxidant	Trigonelline	Flavonoid	Phenol	4-hydroxyisoleucine	Sucrose	Glucose	Fructose
Genotype	39	311.8^**^	0.00084^**^	0.69^**^	0.081^**^	1278^**^	2.47^**^	29.33^**^	177.4^**^
Rep	2	33.28	0.00003	0.0004	0.017	4.92	0.22	2.66	29.2
Error	78	13.67	0.00001	0.003	0.009	14.6	0.047	0.42	4.37
CV (%)	-	4.9	7.27	3	8.75	8.33	21	13.2	8.9

**: Significant at 1% level

The antioxidant activity of the forty Iranian *T. monantha* ecotypes varied from 48.19 to 86.85%, with the highest activity observed in the Tm18, Tm33, and Tm8 ecotypes (Table 4). Conversely, the lowest percentage of antioxidant activity was observed in the Tm39 ecotype. The high antioxidant activity of some ecotypes could be attributed to their phenolic and flavonoid components [38]. Previous studies have shown that the DPPH free radical scavenging power of fenugreek at a concentration of 2 mg/ml is comparable to that of BHT and vitamin C standards [39]. Antioxidant activity plays a crucial role in mitigating the generation of free radicals. Akbari et al. [40] reported that the seed oil of the plant exhibited strong antioxidant radical scavenging activity against the DPPH. Additionally, Naidua et al. [41] identified the plant husk as a valuable source of phenolic acids and a potential natural antioxidant. The antioxidant activity of different plants is primarily related to their phenolic and flavonoid compounds [26, 42].

**Table 4:** The result of means ± standard errors of the measured characters of 40 *T. monantha* ecotypes

Code	Antioxidant (%)	Trig (mmol/l)	Flavonoid (mg quercetin per g dry weight)	Phenol( mg gallic acid per g dry weight )	4-hydro (mg/g)	Sucrose (mM)	Glucose (mM)	Fructose (mM)
Tm1	76.84 ± 0.52	0.056	1.7 ± 0.06	1.04 ± 0.21	0.27 ± 0.018	1.19 ± 0.1	5.46 ± 0.35	26.29 ± 0.42
Tm2	59.68 ± 2.09	0.051	1.94 ± 0.12	0.93 ± 0.12	0.48 ± 0.024	0.35 ± 0.02	9.58 ± 0.35	23.46 ± 0.35
Tm3	58.01 ± 1.92	0.046	1.31 ± 0.13	0.93 ± 0.11	0.26 ± 0.015	0.23 ± 0.02	2.34 ± 0.06	16.5 ± 0.5
Tm4	79.23 ± 0.94	0.054	1.31 ± 0.22	1.1 ± 0.1	0.74 ± 0.023	1.62 ± 0.14	4.88 ± 0.63	22.53 ± 1.19
Tm5	64.35 ± 1.69	0.065	1.74 ± 0.34	1.07 ± 0.09	0.26 ± 0.017	0.18 ± 0.03	1.84 ± 0.1	20.72 ± 1.61
Tm6	66.48 ± 1.59	0.034	1.47 ± 0.26	0.96 ± 0.08	0.26 ± 0.012	2.63 ± 0.2	12.1 ± 0.65	45.55 ± 2.68
Tm7	70.08 ± 1.37	0.045	1.07 ± 0.22	1.05 ± 0.05	0.29 ± 0.017	1.87 ± 0.24	3.78 ± 0.3	20.12 ± 0.77
Tm8	86.29 ± 1.59	0.045	2.19 ± 0.09	1.33 ± 0.15	0.27 ± 0.032	0.24 ± 0.02	1.71 ± 0.09	15.43 ± 0.6
Tm9	78.9 ± 1.04	0.07	1.39 ± 0.08	1.17 ± 0.32	0.75 ± 0.026	1.72 ± 0.1	5.01 ± 0.44	23.75 ± 0.68
Tm10	74.07 ± 1.75	0.064	1.6 ± 0.18	1.02 ± 0.2	0.52 ± 0.017	0.13 ± 0.02	8.74 ± 0.64	26.2 ± 0.57
Tm11	77.71 ± 2.46	0.078	2.02 ± 0.21	1.24 ± 0.21	0.84 ± 0.017	0.33 ± 0.02	2.96 ± 0.05	20.61 ± 1.12
Tm12	78.11 ± 2.08	0.055	1.48 ± 0.42	0.92 ± 0.19	0.25 ± 0.015	2.25 ± 0.15	11.65 ± 0.77	41.71 ± 3.69
Tm13	66.05 ± 2.04	0.072	1.89 ± 0.23	1.34 ± 0.08	0.46 ± 0.019	2.73 ± 0.2	6.05 ± 0.49	26.57 ± 0.33
Tm14	76.21 ± 1.61	0.037	2.68 ± 0.21	0.92 ± 0.17	0.2 ± 0.018	0.57 ± 0.02	4.66 ± 0.38	15.79 ± 0.51
Tm15	73.54 ± 2.61	0.076	2.33 ± 0.27	1.51 ± 0.28	0.87 ± 0.018	0.37 ± 0.04	8.21 ± 0.58	28.97 ± 0.51
Tm16	85.78 ± 2.45	0.065	2.2 ± 0.18	1.06 ± 0.12	0.34 ± 0.018	0.15 ± 0.04	2.06 ± 0.09	19.91 ± 0.35
Tm17	84.87 ± 2.32	0.043	1.54 ± 0.26	1.11 ± 0.16	0.26 ± 0.017	3.77 ± 0.31	9.25 ± 0.7	42.17 ± 2.9
Tm18	86.85 ± 1.61	0.034	2.39 ± 0.32	1.14 ± 0.13	0.25 ± 0.019	0.29 ± 0.03	1.52 ± 0.06	18.77 ± 0.41
Tm19	81.58 ± 1.81	0.023	1.77 ± 0.08	0.88 ± 0.02	0.59 ± 0.029	1.65 ± 0.25	7.71 ± 0.51	29.45 ± 0.48
Tm20	85.99 ± 1.6	0.031	1.65 ± 0.04	0.82 ± 0.04	0.46 ± 0.023	0.85 ± 0.05	5.78 ± 0.42	25.6 ± 0.82
Tm21	74.68 ± 1.32	0.077	2.39 ± 0.11	1.17 ± 0.17	0.71 ± 0.033	0.87 ± 0.04	1.63 ± 0.07	13.39 ± 0.8
Tm22	84.15 ± 1.47	0.035	2.34 ± 0.31	0.95 ± 0.07	0.31 ± 0.009	0.42 ± 0.02	1.48 ± 0.05	15.96 ± 1.2
Tm23	80.22 ± 2.06	0.036	2.35 ± 0.16	0.89 ± 0.09	0.3 ± 0.018	0.41 ± 0.03	1.56 ± 0.07	16.13 ± 1.21
Tm24	84.54 ± 2.59	0.064	1.75 ± 0.18	0.94 ± 0.11	0.91 ± 0.02	1.49 ± 0.21	8.58 ± 0.54	33.47 ± 0.46
Tm25	85.9 ± 2.53	0.071	1.49 ± 0.08	1.03 ± 0.06	0.6 ± 0.035	0.19 ± 0.02	1.07 ± 0.04	15.35 ± 1.23
Tm26	81.23 ± 1.1	0.078	1.58 ± 0.17	1.03 ± 0.13	0.59 ± 0.04	0.17 ± 0.03	1.15 ± 0.08	15.77 ± 0.67
Tm27	82.73 ± 1.9	0.076	1.57 ± 0.15	1.07 ± 0.21	0.59 ± 0.032	0.19 ± 0.03	1.1 ± 0.04	16.42 ± 1.14
Tm28	65.43 ± 2.92	0.05	1.32 ± 0.2	0.92 ± 0.18	0.27 ± 0.012	0.22 ± 0.02	2.45 ± 0.21	16.23 ± 0.99
Tm29	80.77 ± 1.65	0.046	2.39 ± 0.22	1.23 ± 0.19	0.24 ± 0.02	2 ± 0.25	5.31 ± 0.63	26.54 ± 1.44
Tm30	85.65 ± 1.9	0.045	2.17 ± 0.19	0.98 ± 0.09	0.31 ± 0.021	0.68 ± 0.03	9.15 ± 0.35	31.7 ± 1.13
Tm31	83.03 ± 4	0.057	1.66 ± 0.23	0.91 ± 0.08	0.36 ± 0.023	0.41 ± 0.03	6.31 ± 0.1	19.46 ± 1.19
Tm32	83.27 ± 2.79	0.047	1.67 ± 0.14	1.02 ± 0.09	0.29 ± 0.021	0.38 ± 0.01	5.85 ± 0.16	22.54 ± 1.3
Tm33	86.47 ± 1.32	0.066	2.1 ± 0.16	0.86 ± 0.11	0.62 ± 0.024	0.31 ± 0.04	1.26 ± 0.02	16.29 ± 0.78
Tm34	74.92 ± 1.87	0.082	2.48 ± 0.18	1.15 ± 0.12	0.44 ± 0.02	0.91 ± 0.03	5.79 ± 0.44	26.73 ± 1.12
Tm35	74.87 ± 2.2	0.056	2.59 ± 0.19	0.96 ± 0.07	0.82 ± 0.02	1.94 ± 0.29	8.05 ± 0.58	32.37 ± 1.22
Tm36	55.9 ± 1.89	0.081	2.53 ± 0.21	1.3 ± 0.28	0.51 ± 0.009	1.82 ± 0.03	5.29 ± 0.4	23.35 ± 1.17
Tm37	55.14 ± 2.32	0.084	2.52 ± 0.22	1.36 ± 0.23	0.48 ± 0.023	1.94 ± 0.11	4.97 ± 0.57	23.94 ± 1.53
Tm38	63.04 ± 2.59	0.054	3.11 ± 0.28	1.25 ± 0.22	0.65 ± 0.03	1.61 ± 0.3	3.72 ± 0.32	23.01 ± 1.7
Tm39	48.19 ± 1.97	0.076	2.55 ± 0.19	1.29 ± 0.23	0.49 ± 0.012	1.89 ± 0.11	5.92 ± 0.56	24.44 ± 1.5
Tm40	75.84 ± 5.25	0.036	2.43 ± 0.22	0.9 ± 0.07	0.3 ± 0.018	0.39 ± 0.02	1.39 ± 0.08	17.99 ± 1.14
LSD(0.05)	6.01	0.00	0.095	0.15	0.062	0.35	1.05	3.4

Trig: Trigonelline; 4-hydro: 4-hydroxyisoleucine

**Fig. 1:**
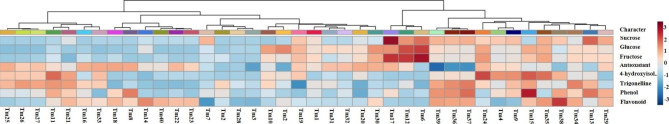
Heatmap clustering of the measured characters of 40 *T. monantha* ecotypes. The color scales represent the values were normalized by Z-score ((value-mean value)/standard error) for each character

**Table 5:** The mean values ± standard errors of the measured characters based on the four groups obtained from the cluster analysis (Fig. 1)

Group	Antioxidant (%)	Trig (mmol/l)	Flavonoid (mg quercetin per g dry weight)	Phenol (mg gallic acid per g dry weight)	4-hydro (mg/g)	Sucrose (mM)	Glucose (mM)	Fructose (mM)
G1	82.07 ± 1.81	0.073	1.91 ± 0.14	1.07 ± 0.05	0.61 ± 0.059	0.32 ± 0.1	1.6 ± 0.28	16.82 ± 1.02
G2	76.53 ± 2.35	0.045	1.89 ± 0.11	0.98 ± 0.03	0.331 ± 0.03	0.58 ± 0.13	4.52 ± 0.73	21.02 ± 1.27
G3	76.49 ± 6.2	0.044	1.5 ± 0.03	1 ± 0.07	0.25 ± 0.004	2.88 ± 0.53	11 ± 1.02	43.14 ± 1.4
G4	69.59 ± 3.9	0.068	2.24 ± 0.18	1.22 ± 0.05	0.61 ± 0.069	1.67 ± 0.19	5.98 ± 0.51	26.31 ± 1.21

Trig: Trigonelline; 4-hydro: 4-hydroxyisoleucine

**Table 6:** The PCA based on the measured characters of 40 *T. monantha* ecotypes

Label	Character	Principal components		
		PC1	PC2	PC3
1	Fructose	0.95	-0.06	0.09
2	Glucose	0.91	-0.06	0.10
3	Sucrose	0.84	0.16	-0.12
4	Trigonelline	-0.13	0.82	0.32
5	Phenol	0.06	0.83	-0.16
6	4-hydroxyisoleucine	-0.002	0.58	0.67
7	Antioxidant	-0.21	-0.52	0.49
8	Flavonoid	-0.16	0.45	-0.52
-	Eigenvalue	2.53	2.20	1.14
-	% of variance	31.68	27.53	14.23
-	Cumulative%	31.68	59.20	73.43

**Fig. 2:**
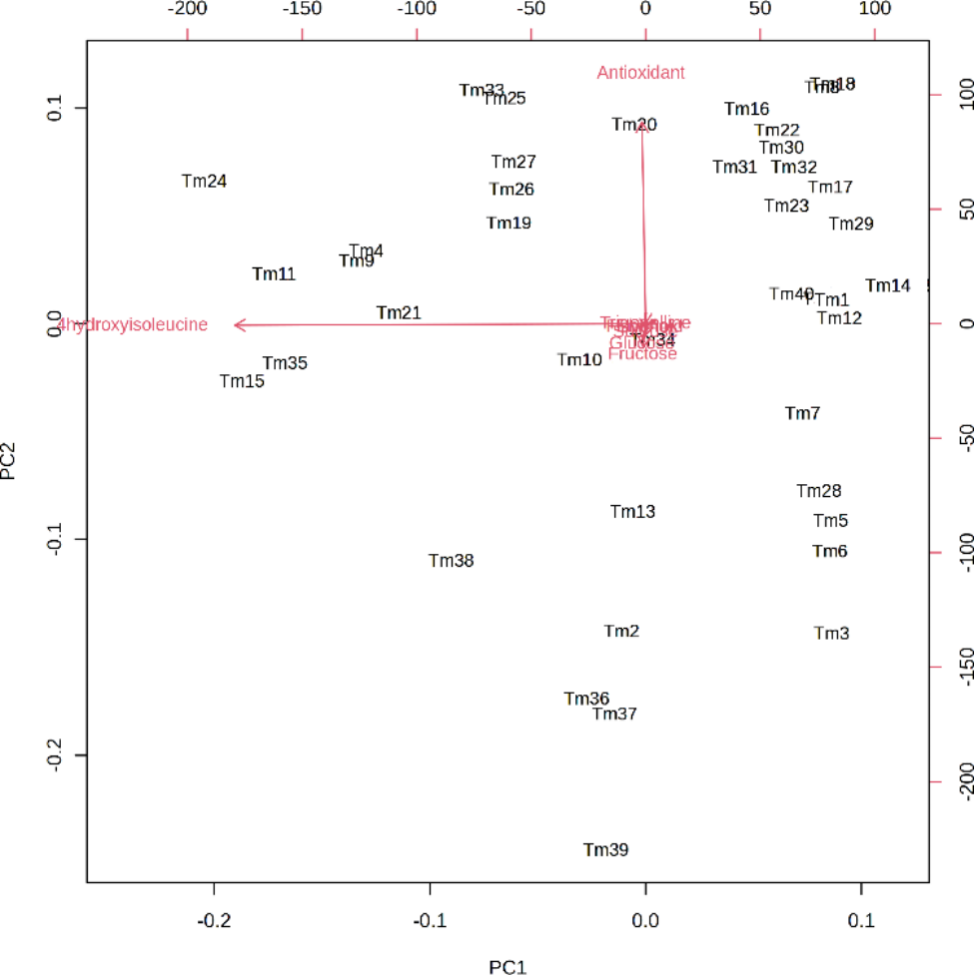
Principal Component Analysis (PCA) biplots of the measured characters of 40 *T. monantha* ecotypes. PC1 explains 31.68% and PC2 27.53% of the variation in the data

**Fig. 3:**
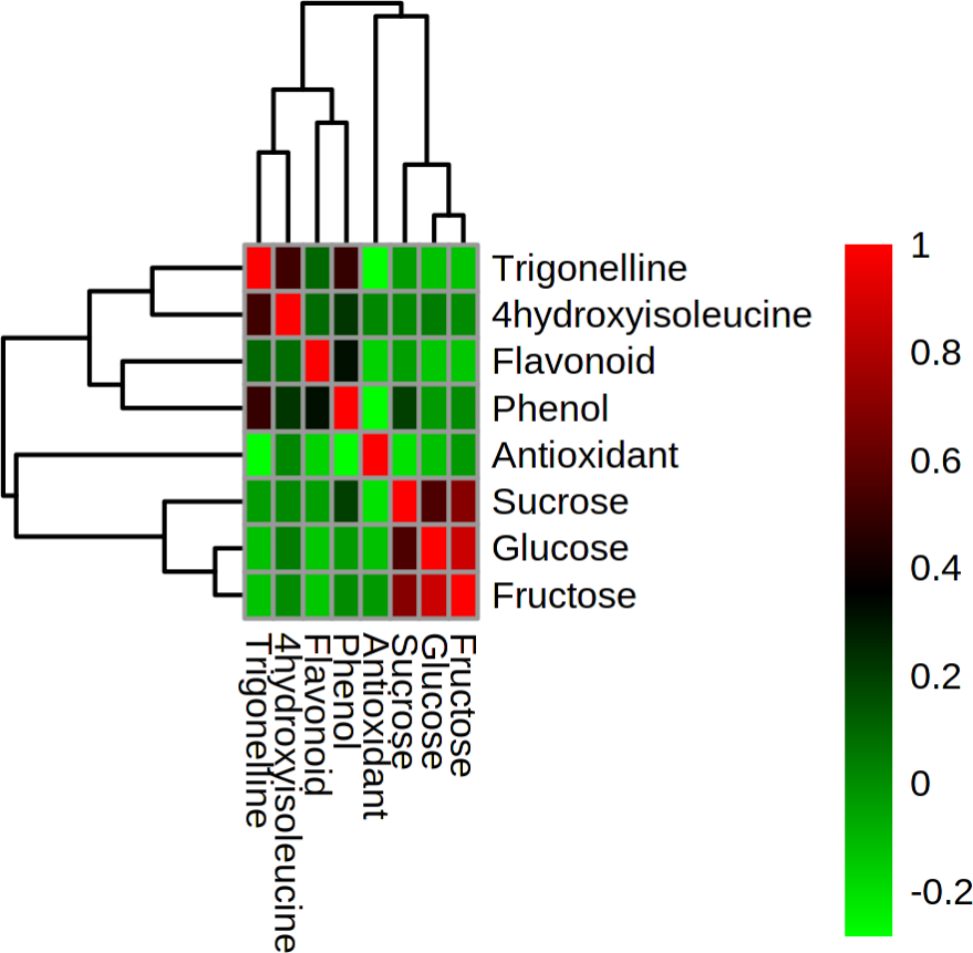
Heat map correlation among the measured characters of 40 *T. monantha* ecotypes

The present study revealed that the highest and lowest phenol contents were observed in Tm15 and Tm 20 ecotypes, respectively. In a previous study, Ali et al. [43] reported a range of 1.27 to 1.39 mg/g of phenol contents in Omani fenugreek, while in this study, the range was between 0.82 and 1.51 mg/g. Moreover, Souri et al. [44] reported a higher phenol content of 1.94 mg/g in the seed of the plant compared to the highest value in the present study. Besides genetic and environmental factors, differences in plant age, harvesting time, storage conditions, processing methods, and the choice of solvent for extraction can also contribute to the variability in phenol content reported in different studies [45].

In a previous study by Joshi et al. [46], the total phenol content of fenugreek varieties under semi-arid conditions was reported to be in the range of 38 to 41 mg gallic acid per g dry weight, and the total flavonoid content was reported to be in the range of 1.2 to 2.3 mg quercetin per g dry weight. However, in our present study, the flavonoid content ranged from 1.07 to 3.11 mg quercetin per g dry weight. Dixon and Paiva [47] reported that environmental factors can significantly influence the variation in flavonoid and phenol content among different plant genotypes through various evolutionary mechanisms. Therefore, it is possible that the observed variations in flavonoid and phenol content among different plant genotypes in our study could be attributed to genetic factors that have been shaped by evolutionary mechanisms, which were influenced by the environment. Huang et al. [48] also reported that the variations in flavonoid and phenol content among different plant genotypes could be attributed to genetic factors that have been influenced by evolutionary mechanisms.

Environmental factors can influence the chemical composition of plants and the synthesis of different compositions with antioxidant potential in different regions [49]. Fenugreek, coffee, soybeans, and peas are some of the plants that are rich in trigonelline components, which is an alkaloid compound formed by the methylation of nicotinic acid [50]. Among the forty Iranian *T. monantha* ecotypes, the highest amount of trigonelline was observed in Tm37 (0.084 mmol/l) ecotype, followed by Tm34 (0.082 mmol/l) and Tm36 (0.081 mmol/l) ecotypes, while the lowest amount was obtained for Tm19 ecotype (0.023 mmol/l). Lalemi and Naghavi [51] reported that the amount of trigonelline in seven *T. monantha* ecotypes ranged from 225 to 819 mg/kg DW. Dry powdered wild fenugreek seeds can contain up to 12% of trigonelline, which has been reported to possess various medicinal properties, including anticancer, anti-migraine, antiseptic, lipid-lowering, and antidiabetic properties [50]. Trigonelline has also been shown to decrease blood glucose levels by inhibiting the activity of key enzymes in glucose metabolism. The ecotypes collected from Rudmajan, Khorasanjonobi (Tm36, Tm37, and Tm39) had a higher level of trigonelline, suggesting that the environmental conditions prevailing at the collection site may have an effect on the component. Banakar et al. [52] demonstrated that the trigonelline content of seeds increased with increasing salinity up to a certain threshold, beyond which further increase in salinity resulted in a reduction in trigonelline levels.

The most abundant free amino acid in fenugreek plant, 4-hydroxyisoleucine, is obtained from the seed endosperm [53]. Among the ecotypes studied, Tm24 (Lorestan, Aleshtar, Kahman village) and Tm15 (Central, Salafchegan to Saveh) exhibited the highest levels of this amino acid, measuring 0.906 and 0.868 mg/g, respectively, while the lowest level (0.197 mg/g) was detected in Tm14 (Markazi, Salafchegan). The literature reports different concentrations of 4-hydroxyisoleucine in fenugreek seeds ranging from 0.015 to 0.4% [32, 54, 55]. Haeri et al. [16] reported higher concentrations of this substance in the seeds from northeastern Iran, with the germinated seeds showing approximately twice the concentration compared to non-germinated seeds. However, there was no relationship between the location of the samples and the concentration of 4-hydroxyisoleucine in the present study. Notably, the highest concentrations of this substance were found across ecotypes collected from various regions, including the south, center, and north of the country. However, Rajabihashjin et al. [56] showed that temperature and solar irradiation contributed prominently to 4-hydroxyisoleucine accumulation. The divergent findings between the present and prior studies may be attributed to the source of the seeds utilized; while the seeds used in the present study were obtained from cultivation, those used in the previous study were sourced from their original sites. Three simple sugars, glucose, fructose, and sucrose, were measured in the Iranian fenugreek ecotypes. Glucose and fructose are monosaccharides, while sucrose is a disaccharide composed of both monosaccharides [57]. Tm6, Tm16, and Tm17 ecotypes, originating from the east, center, and south of the country, respectively, exhibited the highest mean concentrations of the three simple sugars. Navarro del Hierro et al. [58] reported that the major detected carbohydrates in fenugreek plant were sugar alcohols and sucrose. The major abundant sugar alcohols in the plant were d-pinitol and α-d-galactosides of d-pinitol and galactosides of myo-inositol [59]. Sucrose was one of the major soluble carbohydrates in the fenugreek seed (6.41 mg/g DW) [59]. The range of sucrose content in the ecotypes was 0.13 to 3.77 mM. The sucrose content of the studied ecotypes was lower than that reported in a prior study, which suggests that the seeds used in the present study were of superior quality. Furthermore, the concentrations of glucose (1.07 to 12.1 mM) and fructose (13.3 to 45.5 mM) were higher than the sucrose in the plant seeds. Our body absorbs glucose and fructose more easily than sucrose, and sucrose absorption causes a rise in blood sugar resulting in the rupture of blood vessels and mouth problems [60]. Therefore, the ecotypes with less sucrose and higher levels of glucose and fructose, such as Tm6 and Tm12, had better quality. The present study did not find a relationship between the location and the three soluble carbohydrates. However, Aljuhaimi et al. [61] reported that the sugar compositions of fenugreek seeds changed based on locations.

Cluster analysis was performed to classify the ecotypes based on the measured traits, and the results are presented in Fig. 1. The ecotypes were grouped into four distinct clusters. The first cluster comprised seven ecotypes, including Tm25, Tm26, Tm27, Tm11, Tm21, Tm16, and Tm33. These ecotypes exhibited the highest antioxidant activity and trigonelline content (Table 5), while displaying the lowest values for glucose, fructose, and sucrose. These ecotypes were collected from various regions across the country. The second cluster consisted of 18 ecotypes, exhibiting moderate values for most of the characteristics, except for phenol content (0.98 mg gallic acid per g dry weight), which was the lowest among all the clusters. Ecotypes Tm6, Tm12, and Tm17 were grouped in the third cluster and had the highest values for glucose, fructose, and sucrose, but the lowest levels of antioxidant activity, trigonelline, flavonoid, and 4-hydroxyisoleucine. The fourth cluster comprised 12 ecotypes, exhibiting the highest mean values for flavonoid, phenol, and 4-hydroxyisoleucine contents. The study revealed significant variation in the measured traits among the ecotypes, which could be attributed to genetic factors and their interaction with the environment. The samples were cultivated under the same experimental conditions, and no correlation was found between the sample location and the level of each trait. Acharya et al. [62] reported that the production of phytochemical components in the plant seed by similar genotypes could vary significantly across different locations due to the strong genotype X environment (GE) effect. The results of this study further supported this finding, as ecotypes that exhibited high levels of certain characteristics at one location did not necessarily produce the same level of those characteristics at another site. For instance, ecotype Am17 had high levels of the three simple sugars, while Am18 collected from the same location exhibited lower levels of these sugars. The environmental factors had a lesser effect on the total variations observed in some characteristics of the plant seed, such as diosgenin content [63]. The study also reported that the main effect of the environment contributed the highest to the total variation (78%) of 4-hydroxyisoleucine content.

Principal component analysis (PCA) was performed based on the measured characters of 40 *T. monantha* ecotypes. The first three principal components (PC1, PC2, and PC3) explained more than 73% of the total variance among the ecotypes (Table 6; Fig. 2). PC1 explained 31.68% of the total variance and showed a positive correlation with three simple sugars: glucose, fructose, and sucrose. The heat map correlation (Pearson correlation coefficient) revealed a strong positive correlation among the three simple sugars (Fig. 3), forming a cluster with antioxidant activity appearing in a separate cluster. Phenol, flavonoid, trigonelline, and 4-hydroxyisoleucine formed another cluster, positively correlating with the second PC, while antioxidant activity negatively correlated with this PC. PC2 and PC3 explained more than 27 and 14% of the total variance, respectively.

This research is a significant step towards the identification and selection of Iranian wild fenugreek ecotypes as a source of natural health products, highlighting the high level of seed chemical diversity among these ecotypes. Some of these ecotypes have a high level of chemical composition that could potentially be useful for industrial applications and serve as valuable germplasm for human nutrition and breeding programs.

## Data Availability

All data are within the manuscript.
